# The Relationship between Parental Educational Involvement and Learning Engagement among Chinese Middle School Students: The Mediating Effect of Gratitude and Hope

**DOI:** 10.3390/bs14080687

**Published:** 2024-08-08

**Authors:** Fang Chen, Jinhong Wang, Wenyuan Zhang, Peijuan Li, Yadi Zeng, Hongyu Zou

**Affiliations:** 1 College of Education and Sports Sciences, Yangtze University, Jingzhou 434023, China; 2 School of Psychology, South China Normal University, Guangzhou 510631, China; 3 Department of Public Basic Courses, Changjiang Polytechnic of Art and Engineering, Jingzhou 434000, China; 4 School of Psychology, Shaanxi Normal University, Xi’an 710062, China

**Keywords:** middle school students, parental educational involvement, gratitude, hope, learning engagement

## Abstract

Despite the extensive body of literature on the correlation between family dynamics and academic achievement among students, there remains a notable gap in research investigating the influence of parental educational involvement on student learning engagement. Based on the developmental–ecological model of student engagement and relevant theoretical frameworks, this study used the quantitative analysis method to construct a chain mediation model to test the relationship between parental educational involvement and the learning engagement of middle school students, as well as the potential mediating role of gratitude and hope. This study employed a cross-sectional study using whole-cluster random sampling to measure middle school students aged 11–18 years old in two schools over a one-week period. Through the use of self-reporting surveys, this study assessed the levels of parental educational involvement, gratitude, hope, and learning engagement among 754 middle school students (48% female; *M*_age_ = 14.83, *SD* = 1.65) in Hubei Province, China. The mediation effect was analyzed using regression analysis and the chained mediation model and tested via the Bootstrap method. The findings suggested that parental educational involvement significantly positively related to learning engagement among middle school students, while gratitude and hope serve as partial mediators in the relationship between parental educational involvement and learning engagement. These findings revealed the psychological mechanisms underlying the relationship between parental educational involvement and learning engagement among middle school students, providing valuable insights for enhancing their level of learning engagement.

## 1. Introduction

Enhancing parental involvement in students’ educational process is a significant focus of current educational research. Numerous studies have revealed that parental educational involvement can positively relate to students’ academic outcomes [1,2]. Learning engagement, which reflects a positive student status, has also received extensive attention in educational research and practice. It fosters an ongoing positive state during learning activities, motivating students to strive for excellence [3,4]. Research has found that various factors impact students’ learning engagement, leading to many positive outcomes, such as helping students complete their studies and promoting mental health and future development [5]. Among these factors, parental educational involvement is crucial for enhancing students’ learning engagement [6,7]. Middle school education plays a vital role in fostering the pursuit of knowledge and holistic development. Increasing students’ learning engagement during this critical period is an effective strategy for ensuring quality education and fostering students’ growth and success in modern society.

Despite the fruitful research related to family factors on middle school students’ learning engagement, there is a lack of studies focusing specifically on the relationship between parental educational involvement and learning engagement and its internal mechanisms. The developmental–ecological model of student engagement [8] suggests that personal dispositions and social environment play crucial roles in the learning and development of students, with the support of significant individuals like parents, teachers, and peers being particularly influential in optimizing students’ motivation to learn. This study aims to investigate the impact of parental educational involvement on middle school students’ learning engagement and to explore the underlying psychological processes related to gratitude and hope.

### 1.1. Parental Educational Involvement and Student Learning Engagement

Parental educational involvement encompasses a variety of strategies utilized by parents to support their children’s academic achievements, such as emotional, behavioral, and intellectual involvement [9]. According to ecosystem theory [10], families, as a microsystem, have the most direct influence on an individual’s psychology and behavior. Past studies have shown that parental involvement is crucial for students’ academic performance, helping them adapt to the school environment and participate in learning activities. For example, in Fan and Williams’ study, parental expectations for their children’s education, positive school–parent interactions, and the provision of guidance and advice by parents were positively associated with 10th-grade students’ academic self-efficacy, intrinsic motivation to learn, and levels of engagement in learning, primarily in English and Math [11]. Kurt and Tas’s study showed that aspects of parental educational involvement, such as parental engagement in school activities, educational willingness, parent–child communication, and autonomy support, can meet the basic psychological needs of Turkish students and facilitate their motivation and engagement in learning [12]. In China, parents have always attached great importance to their children’s educational involvement and expect their children to have excellent learning status and academic achievements. However, there are fewer studies related to the effect of parental educational involvement on students’ learning engagement, and they are mainly focused on the primary school level; there is a lack of research on middle school students. Liu et al. concluded that the higher the perceived parental educational involvement of primary school students, the more likely they are to generate support for achievement goal orientation and the stronger their level of academic self-efficacy, which further leads to higher levels of academic engagement [13]. The family investment model [14] suggests that the more resources (both material and emotional) parents put into their children’s education, the more beneficial it is for the positive academic and cognitive development of adolescents [15]. The direct or indirect active participation of parents in providing extra educational resources to their children, staying updated on their children’s school performance, and offering timely support and encouragement in times of learning difficulties can effectively boost their motivation and engagement in learning [16]. In conclusion, these studies present proof that parental educational involvement generally has a positive effect on students’ learning engagement. Thus, we assumed that parental educational involvement may be positively related to middle school students’ learning engagement (H1).

### 1.2. Gratitude as a Mediator

Parental educational involvement can impact adolescents’ learning engagement by influencing internal individual factors, with gratitude and hope being significant psychological factors. In both Western religion and philosophy and traditional Chinese culture, gratitude is recognized as a positive virtue and trait. In psychology, gratitude is often explained as an emotion directly related to receiving support from others that promotes positive reactions to experiences and outcomes [17,18]. Given the meaning and character of gratitude, it is clear that perceived positive experiences and outcomes are important prerequisites for gratitude. Parental involvement plays a fundamental role in developing gratitude by supporting children’s growth and learning. Research has demonstrated that parental involvement in education sets the stage for fostering gratitude by supporting individuals in their physical and mental growth and learning processes [19,20]. In addition, the family is the first place where children’s values and emotional attitudes are formed and the family crucial for the formation and development of gratitude. The examples above provide indirect evidence for us to better comprehend the relationship between parental educational involvement and students’ learning engagement.

Numerous studies have found that the quality of gratitude predicts students’ learning engagement. The positive effects of gratitude can be reflected in students’ behaviors when performing academic and non-academic activities, which promote engagement in academic pursuits [21]. A study by Valdez et al. found that a Facebook-based online gratitude intervention increased students’ perceived social connectedness, positive thoughts, and tendency to give back to others among Filipino high school students, positively affecting academic motivation and engagement outcomes [22]. Meanwhile, a nine-week gratitude journal intervention study among Japanese high school students found that sustained feelings of gratitude had a protective effect against declining academic motivation and a significant impact on goal-directed behavior [23]. That is, the development of gratitude may have a positive impact on their learning motivation and emotions. Additionally, Jin and Wang found that gratitude can enhance positive impacts on learning engagement by facilitating the optimization of teacher–student relationships and satisfy the psychological needs of Chinese junior high school students [24]. All of these findings suggest that gratitude can catalyze student motivation and engagement in learning through a range of cognitive, affective, and interpersonal mechanisms. According to the broaden and build theory of gratitude, gratitude not only expands cognitive processes but also nurtures expressions of affection and creative ideation, potentially leading to enduring positive tendencies [25]. In Chinese educational culture, students often express gratitude through academic pursuits, showing increased enthusiasm for learning activities when supported by parents. This active involvement suggests that gratitude may mediate the relationship between parental educational involvement and learning engagement [26,27]. Therefore, we hypothesized that gratitude may mediate the relationship between parental educational involvement and students’ learning engagement (H2).

### 1.3. Hope as a Mediator

Hope plays a crucial role in an individual’s development, and it is influenced by personal traits, social interactions, and other factors [28]. Described as a positive motivational mindset, hope involves cognitive processes that steer individuals toward their aspirations, blending cognition and emotion to promote self-growth and enhance feelings of proficiency [29]. According to hope theory [29], hopeful individuals, especially children, are guided toward their goals through positive support and role modeling (e.g., parents, teachers, etc.). Previous studies have shown that parental involvement, particularly in education, can offer significant support and motivation to individuals, teaching them the importance of setting and striving for goals [30,31]. Appropriate parental involvement can enhance the psychological strength of Chinese middle school students to promote the development of hope [32]. Specifically, parental involvement in emotional and behavioral aspects can contribute to students having a positive mindset and motivation to pursue their goals in a planned and purposeful manner. Meanwhile, research has indicated that hopeful students demonstrate high levels of motivation, confidence, and effective learning strategies, contributing to their optimism for the future and success in learning [29,33]. Tomás et al. argued that hope refers to a positive perception of the future and involves determination to achieve goals [34]. Their study found that hope in Dominican Republic middle school students was positively correlated with behavioral, affective, and cognitive engagement in learning. For middle school students, hope facilitated students in terms of planning their learning and flexibly applying cognitive strategies to achieve their academic goals, as well as feeling optimistic and excited about the future. Then, students are more motivated, more engaged in learning, and more likely to achieve academic success [35,36]. Therefore, we hypothesized that hope may mediate the relationship between parental educational involvement and students’ learning engagement (H3).

### 1.4. The Chain-Mediating Role of Gratitude and Hope

According to the family investment model [14], families that actively participate in their children’s education consistently provide various developmental resources, including material support, human support, and emotional resources. These resources are essential in meeting the fundamental psychological needs of middle school students, which, in turn, contribute to the development of positive traits. Gratitude and hope are characterized by positive emotional attributes that can either be temporary states (e.g., feeling grateful or hopeful in the moment) or enduring personality traits (e.g., consistently demonstrating gratitude or hopefulness). So, what is the connection between gratitude and hope? Taruna et al.’s study found a positive correlation between gratitude and hope and affirmed the positive effects of both on adolescent mental health [37]. Zhang et al.’s study discovered that negative life events reduce rural adolescents’ hope and their ability to self-regulate and problem-solve, thus impairing the production and expression of gratitude [38]. This result may be attributed to viewing hope and gratitude as two separate traits or states and focusing more on their aftereffects. It is important to note that gratitude and hope have different orientations in time. Gratitude is focused on the past (expressing appreciation for support received), while hope looks toward the future (expecting positive outcomes) [39]. According to the temporal dimension of the positive psychological traits model, the past psychological trait of gratitude influences the present and future psychological trait of hope [40]. Research conducted by McCullough et al. suggests that practicing gratitude enhances hope in individuals, leading them to be more proactive in their lives [41]. Moreover, an experiment conducted by Witvliet et al. [42] instructed participants to reflect on past experiences and create a narrative about a significant event, emphasizing sources of gratitude and appreciation. The results showed that recalling grateful memories increased participants’ levels of hope and overall happiness. Accordingly, we consider that higher levels of parental involvement in a child’s education fosters feelings of respect, understanding, and support in the child, potentially leading to the development of gratitude and related behaviors. This, in turn, boosts motivation and determination in pursuing goals. Individuals with high levels of hope are more likely to achieve their objectives through their beliefs, cognitive abilities, and motivation. They approach academic challenges with optimism, proactively work toward their goals, and actively engage in the learning process. Therefore, we hypothesized that perceived social support and hope play a chain-mediating effect in the relationship between parental educational involvement and students’ learning engagement among middle school students (H4).

### 1.5. The Present Study

**Figure 1:**
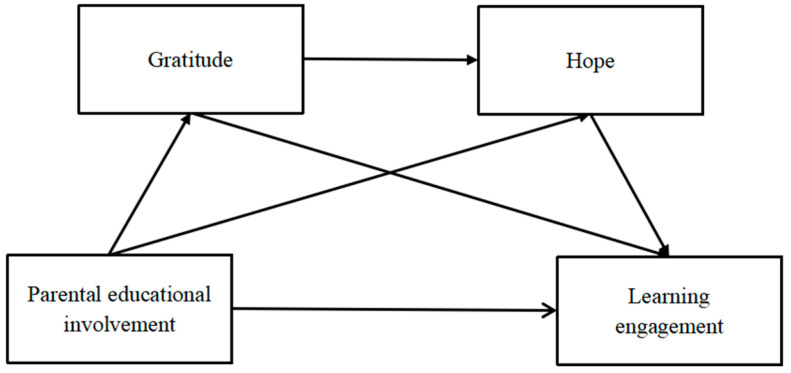
The proposed moderated mediation model.

Although no research has directly demonstrated the chain-mediating role of gratitude and hope in the relationship between parenting involvement and students’ learning engagement, based on the literature review above, we learned that the variables in this study are interrelated. Based on the theoretical frameworks and related research of the developmental–ecological model of student engagement and the family investment model [8,14], students’ learning behaviors are subject to multiple influences, such as individual psychology and the external environment. Meanwhile, gratitude and hope may play a bridging role in the relationship between parental educational involvement and students’ learning engagement [19,24,28,43]. Hope theory and the broaden and build theory of gratitude also support the positive effects of gratitude and hope on learning engagement [25,29]. However, few studies have incorporated both gratitude and hope into research models. In conjunction with empirical research on the temporal dimension of the positive psychological traits model [40], gratitude is believed to promote hope, and the emotion and disposition of gratitude promotes students’ motivation and enthusiasm for learning and being hopeful. Therefore, this study will test a chain mediation model to examine the association between parental educational involvement and students’ learning engagement among middle school students, while also analyzing the mediating effects of gratitude and hope in an integrative way. The proposed model is illustrated in Figure 1.

## 2. Method

### 2.1. Participants and Procedure

This study employed a cross-sectional study [44] in which questionnaires were administered to middle school students between the ages of 11–18 years old over a one-week period. The questionnaire was administered in the school on a class basis using the whole-cluster random sampling method, with consent sought from both their guardians and main school leaders. The participants were informed that their responses would be used solely for academic research purposes, ensuring adherence to principles of voluntary participation, anonymity, and confidentiality. In each class, a psychology graduate student served as the primary examiner, providing an introduction to the questionnaire’s requirements and precautions, and subsequently distributing the questionnaires to the participants for completion. Self-reported information included a demographic information questionnaire, the Parental Educational Involvement Scale, the Gratitude Questionnaire, The Children’s Hope Scale, and the Learning Engagement Scale. The testing environment was maintained in good condition during the answering process, and personal guidance was provided as necessary to ensure that participants understood each option correctly. All of the students were required to complete the questionnaire within an allotted 30 min prior to on-site collection. After collecting the questionnaires, defaced questionnaires, cases where basically the same option has been filled in, or those with questions left unfinished, were eliminated and considered invalid.

The participants of this study were students from two middle schools located in Hubei Province, China. A total of 803 questionnaires were distributed, out of which 754 valid responses were collected, resulting in an effective response rate of 94.90%. Among the respondents, there were 391 male students (51.86) and 347 female students (48.14%); 401 junior high school students (53.18%) and 353 senior high school students (46.81%); 327 only children (43.37%) and 410 non-only children (56.63%); 269 class cadres (35.68%) and 468 non-class cadres (64.32%); and 407 (53.98%) urban students and 323 (46.02%) rural students. The average age of the participants was recorded as being 14.83 ± 1.65 years. All of the participants aged 18 and older provided written informed consent, while participants under the age of 18 had written consent signed by their guardians (teachers or parents). All of the participants could choose to voluntarily participate or withdraw from the study. The research was conducted in accordance with the Declaration of Helsinki, and the protocol received approval from the Ethics Committee of Yangtze University College of Education and Sports Sciences.

Based on Monte Carlo simulations, we calculated the sample size of the relevant models and inverted the current effect sizes of the models; in this dataset, the minimum sample size of the chained mediation model between parental educational involvement and learning engagement (taking a power value of 0.8) was N = 128; when taking a power value of 1.0, it was N = 254. This study’s sample size exceeded the minimum sample size requirements.

### 2.2. Measures

#### 2.2.1. Parental Educational Involvement

The Parental Educational Involvement Scale, which was a Chinese scale developed by Xia [42] based on the theoretical frameworks proposed by Grolnick and Keith [9], was employed in this study. Such questionnaires have been widely used in most studies focusing on parental educational involvement in China [45,46]. This scale combines the actual situation of parenting in China and the characteristics of the middle school student population and is applicable to the study of the parental educational involvement of Chinese students. In the original study, it proved to have good reliability and validity. In research related to parental involvement, children’s perceptions and attitudes toward parental educational involvement are essential to determining whether parents can effectively influence children [47]. Therefore, this scale provides a comprehensive measure of students’ perceived parental educational involvement through a self-reported measure involving three dimensions: intellectual involvement, emotional involvement, and behavioral involvement (e.g., “My parents communicate with me about school-related matters or activities”). Comprising 18 items rated on a 5-point scale (1 = never; 5 = always), higher scores indicate greater parental educational involvement in their children’s education. In this study, Cronbach’s α was 0.89.

#### 2.2.2. Gratitude

The Gratitude Questionnaire (GQ-6) developed by McCullough et al. was administered [17]. It consists of 6 items scored on a 7-point Likert scale (1 = completely disagree; 7 = completely agree), with questions 3 and 6 being reverse-scored. Higher scores indicate greater levels of gratitude. This scale has been widely used and validated in China [48,49]. In this study, Cronbach’s α was 0.81.

#### 2.2.3. Hope

The Children’s Hope Scale was developed by Snyder et al. [28] and translated and revised by Zhao and Sun for use in China [50]. The scale comprises 6 questions with two dimensions: path thinking and motivational thinking (e.g., “When I am in trouble, I will think of many ways to overcome it”). It is scored on a 6-point Likert scale (1 = strongly disagree; 6 = strongly agree), with higher scores representing stronger hope. In this study, Cronbach’s α was 0.88.

#### 2.2.4. Learning Engagement

The Chinese version of the Learning Engagement Scale was used in this study. It was originally developed by Schaufeli [51] and subsequently revised by Fang et al. [4]. This scale encompasses three dimensions, namely vigor, dedication, and concentration, comprising a total of 17 items (e.g., “I feel energized when I engage in studying”). The responses were rated on a 7-point scale (1 = never; 7 = always), with higher scores indicating greater levels of learning engagement. In this study, Cronbach’s α was 0.96.

### 2.3. Data Analysis

SPSS26.0 and PROCESS were utilized for the data analysis. First, we employed SPSS 26.0 to examine potential methodological biases via a Harman one-way factor test. There are 8 factors with characteristics exceeding 1, with the first factor accounting for 33.02% (<40%), indicating there was no serious common method deviation in this study. Second, descriptive analysis was conducted on demographic information and key study variables, while Pearson correlation analyses were utilized to assess variable correlations. The chained mediation model analysis (Model 6) was subsequently conducted using the PROCESS 3.4 macro program [52], as this model assumes that the mediating variables M1 and M2 can act as both separate and continuous mediators between the independent variable X and the dependent variable Y. The bias-corrected percentile Bootstrap method was used to test for significance with a confidence interval of a 95% bias-corrected percentile. Furthermore, to account for potential confounding factors, the main analysis controlled for gender, age, only child status, place of birth, and being a class representative. This was conducted because previous studies have found these factors to be closely related to the main variables used in this study [34,53].

## 3. Results

### 3.1. Descriptive Statistics

Descriptive statistics were performed for each of the main study variables. The results are shown in Table 1.

### 3.2. Correlation Test of Scale Scores

The main study variables were subjected to correlation analyses, with the results presented in Table 2. Parental educational involvement exhibited a significant positive correlation with middle school students’ gratitude (*r* = 0.42, *p* < 0.001), hope (*r* = 0.42, *p* < 0.001), and learning engagement (*r* = 0.47, *p* < 0.001). Gratitude demonstrated a significant positive correlation with hope (*r* = 0.49, *p* < 0.001) and learning engagement (*r* = 0.51, *p* < 0.001), while hope displayed a significant positive correlation with learning engagement (*r* = 0.56, *p* < 0.001).

### 3.3. Regression Analysis of Parental Educational Involvement, Gratitude, Hope and Students’ Learning Engagement

**Figure 2:**
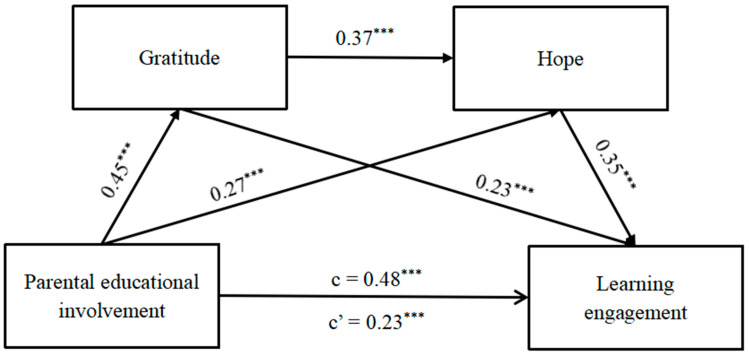
The chain-mediating role of gratitude and hope in the relationship between parental educational involvement and learning engagement. Note. *** *p* < 0.001.

The variables were first standardized, and chained mediation effect analysis was conducted using Model 6 in the macro program PROCESS developed by Hayes. Regression analysis was then performed with parental educational involvement as the independent variable, learning engagement as the dependent variable, and gratitude and hope as mediator variables while also controlling for gender, age, being a single child, being a class cadre or not, and residential area. The results (Table 3 and Figure 2) indicated that before considering the mediator variables, parental educational involvement significantly and positively predicted middle school students’ learning engagement (*β* = 0.48, *t* = 14.41, *p* < 0.001), indicating that the total effect was significant enough to allow for further mediation path analysis. After incorporating gratitude and hope as mediating variables into the model, parental educational involvement still significantly and positively predicted middle school students’ learning engagement (*β* = 0.23, *t* = 6.81, *p* < 0.001). Additionally, parental educational involvement had significant positive effects on both gratitude (*β* = 0.48, *t* = 14.41, *p* <0.001) and hope (*β* = 0.45, *t* = 13.35, *p* < 0.001). Furthermore, gratitude significantly predicted both hope (*β* = 0.37, *t* = 10.86, *p* < 0.001) and learning engagement (*β* = 0.23, *t* = 6.83, *p* < 0.01), while hope significantly positively influenced learning engagement (*β* = 0.35, *t* = 10.41, *p* < 0.001).

### 3.4. Chain-Mediating Effects of Gratitude and Hope

Furthermore, the Bootstrap mediation effect test results (Table 4) revealed significant mediation effects of gratitude and hope in the relationship between parental education involvement and learning investment, comprising three distinct paths. The indirect effect for path 1, “parental educational involvement → gratitude → learning engagement”, was estimated to be 0.10, with a 95% confidence interval [0.07, 0.14]. The indirect effect for path 2, “parental educational involvement → hope → learning engagement”, was found to be 0.09, with a confidence interval of [0.07, 0.14]. The indirect effect for path 3, “parental educational involvement → gratitude → hope → learning engagement”, was determined to be 0.06 (95% CI: [0.04, 0.08]). Importantly, all three paths exhibited statistically significant effects as their respective confidence intervals did not encompass 0. Specifically, gratitude and hope were identified as separate partial mediators, as well as sequential mediators, in the relationship between parental educational involvement and learning engagement. Additionally, three pairs of comparisons revealed statistically significant differences between indirect effect 1 and indirect effect 3, indicating that the separate mediation of gratitude had a higher impact compared to the continuous mediating effect of both gratitude and hope.

## 4. Discussion

### 4.1. The Relationship between Parental Educational Involvement and Middle School Students’ Learning Engagement

This study revealed a positive association between parenting educational involvement and learning engagement among middle school students, which aligns with prior research findings [11]. This finding is also consistent with the developmental–ecological model of student engagement [8], emphasizing the pivotal role parents play within the family unit as primary role models and influencers on their children’s behaviors and attitudes, ultimately exerting a profound impact on their children [10]. In this study, parents who demonstrate a strong emphasis on and active involvement in education are likely to have children with positive attitudes and behaviors toward learning. Specifically, parental involvement in education creates a positive home educational climate that includes encouraging children to learn, providing companionship and comfort in times of difficulty and granting children a degree of autonomy. This positive climate can stimulate children’s interest and engagement in learning [11]. Simultaneously, parental involvement in education means that they are more attentive to their children’s learning and provide positive support and feedback. Parents possess awareness regarding their children’s academic progress, exhibit concern for their performance, and actively assist in the development and realization of learning plans by offering resources and guidance. These engagements serve to cultivate students’ interest in learning while enhancing their problem-solving abilities, thereby promoting increased engagement in the learning process [13]. Moreover, this study did not yield any additional findings regarding the correlation between parental educational involvement and learning engagement among middle school students, which has been explored in previous studies [54,55]. This may be attributed to the lack of differentiation in this portion of this study’s measure of parental involvement between parents’ own educational engagement and children’s perceptions thereof. The effectiveness of parental influence could be contingent upon children’s attitudes and perceptions toward parental involvement [13]. The parenting educational involvement measure utilized in this study entailed self-reported perceptions of parental engagement as reported by middle school students. The findings revealed a positive correlation between increased levels of parental educational involvement and higher learning engagement among middle school students. Thus, middle school students perceived parental educational involvement as a significant protective factor for students’ learning engagement.

### 4.2. The Mediating Role of Gratitude

This study revealed that gratitude serves as a mediating factor in the association between parental educational involvement and academic engagement among middle school students, aligning with our initial hypothesis. In accordance with the concept of gratitude, individuals experiencing appreciation and pleasure upon receiving a favor are motivated by this emotional awareness to reciprocate [17]. This process encompasses both the giver and the receiver; for example, when middle school students perceive that their parents have invested more in their education, they may develop feelings of gratitude and thus repay their parents by investing in their studies and striving to achieve learning outcomes. Parental educational involvement in education positively predicts the gratitude levels of middle school students, which aligns with previous research [56]. Parental involvement in education means that they provide educational resources and support for their children. Children may develop a sense of gratitude toward the opportunities and conditions created by their parents, thereby cultivating and dispositioning toward gratitude [57,58]. Meanwhile, parental involvement in education is often accompanied by positive feedback and encouragement. Parental encouragement and appreciation stimulate gratitude when children make progress and experience achievements [59]. Additionally, gratitude positively predicts learning engagement in middle school students, which is consistent with previous studies [22,23] and in accordance with Fredrickson’s broaden and build theory of gratitude [25]. When middle school students possess an awareness of the opportunities, resources, and help they have from others, they are appreciative. Consequently, they are more inclined toward positive motivations and exhibit favorable attitudes toward learning and are more refreshed to face learning tasks and challenges [17,19]. These points can be exemplified in the following: “A drop of kindness deserves a fountain of gratitude”. Within the cultural context of Chinese filial piety and reciprocation, when middle school students cultivate gratitude toward their parents and experience a sense of appreciation, they exhibit a willingness to diligently pursue academic excellence as a means of reciprocating their parents’ contributions [24]. Consequently, when children perceive their parents’ active involvement in education, they experience a sense of being valued and cared for and may appreciate the support and resources provided by their parents. Such feelings and tendencies of gratitude will prompt children to face learning tasks and challenges with more optimism and enhance self-confidence and motivation in their own learning abilities. Ultimately, these factors contribute to heightened levels of learning engagement. The findings of this study unveil the significant role of affective factors in students’ learning engagement. Learning engagement encompasses not only cognitive processes but also emotional processes [3]. Gratitude, as a positive affective attribute, amplifies students’ motivation and emotions, fostering their affection for and enjoyment of learning [60]. This implies that, in the future, for family and school education, while focusing on the cognitive development of students, we should also pay attention to their emotional experience and cultivate the virtue of gratitude, thereby facilitating the augmentation of learning engagement.

### 4.3. The Mediating Role of Hope

This study revealed that hope serves as a mediator in the association between parental educational involvement and learning engagement among middle school students, thereby supporting our hypothesis. This finding aligns with hope theory [29], which posits that a hopeful outlook in students can be fostered through positive connections and interactions with significant figures in their lives (e.g., parents and teachers). Consequently, they acquire the ability to identify pathways toward their goals, sustain motivation for these objectives, develop optimistic expectations for the future, exhibit confidence, and display heightened levels of learning engagement. The positive predictive impact of parental educational involvement on the hope of middle school students is consistent with previous research findings [61]. Parental involvement in education means that they keep a close eye on the learning and development of their children, provide ample encouragement and support, and impart the significance of goal-setting and pursuit to their children. Middle school students perceive a sense of value and encouragement from this, enabling them to establish connections between academic achievements and their desired objectives, thereby fostering increased hope [31,62]. Furthermore, hope has been found to be a significant and positive predictor of academic engagement, which aligns with previous empirical findings [33]. Hope can foster the development of adaptive and functional strategies among students, thereby stimulating their intrinsic motivation and encouraging proactive learning behaviors [63]. Moreover, hope fosters a sense of purpose and ambition among middle school students; they establish clear learning objectives and exert diligent efforts to attain them. They possess the belief that their endeavors can lead to triumph and hold optimistic expectations [35,36]. This hopeful mindset propels their dedication toward learning, rendering them more focused and industrious [43]. Therefore, hope plays a pivotal role in bridging parental involvement and students’ learning engagement. As motivation driven by external pressures and desires tends to wane over time, it is the internal drive for self-improvement based on personal interests and needs that exerts a lasting motivational impact. Thus, fostering hope encompassing goals, pathways, and intrinsic motivation becomes particularly crucial in promoting middle school students’ active engagement in learning.

### 4.4. The Chained Mediating Effects of Gratitude and Hope

This study also found that gratitude and hope serve as sequential mediators in the relationship between parental educational involvement and learning engagement among middle school students. Specifically, parental educational involvement can influence hope by fostering gratitude, subsequently impacting learning engagement via hope, which aligns with our initial hypothesis. The finding is also in line with the family investment model [14], which posits that parents enhance their children’s positive abilities and psychological resources through active investments, thereby increasing the likelihood of positive developmental outcomes. This observation is also in line with the temporal dimension characteristics of the positive psychological traits model proposed by Seligman [40]. Gratitude involves appreciation for benefits already received, while hope involves a positive anticipation of desired future outcomes [39]. These two constructs follow a sequential order, as optimistic expectations for the future may be built upon feelings of gratitude toward past positive experiences or outcomes [64]. Thus, the emotion of gratitude for positive experiential events subsequently inspires positive beliefs and good expectations about the future, i.e., hope. Furthermore, both gratitude and hope are classified as positive emotions, wherein one emotion can stimulate the other [41]. These theoretical frameworks elucidate the mechanism through which gratitude can elicit feelings of hope. Prior research has also demonstrated that gratitude can facilitate the development of enduring positive psychological resources, bolster resilience, and enhance overall life appraisal [65]. When individuals possess a heightened level of gratitude, they are more inclined to perceive and acknowledge the positives and benefits in their surroundings while cultivating mindfulness and awareness toward the significance of their environment. This may augment their hopefulness for the future [41]. Therefore, when middle school students perceive parental involvement in their education, they will develop a profound appreciation for the love and support bestowed upon them by their parents, thereby engendering a sense of gratitude. This sentiment of gratitude subsequently fosters an inclination among middle school students to value the resources at their disposal, cultivate an optimistic mindset, and nurture positive psychological assets, ultimately augmenting their hope for the future. Consequently, middle school students brimming with hope exhibit heightened motivation and enthusiasm toward learning endeavors, invest greater effort in pursuing educational objectives, and show higher engagement in learning. These findings highlight the importance of not only providing material support and educational resources, but also addressing children’s emotional needs and fostering positive psychological qualities in our involvement in students’ educational process. By cultivating positive emotional qualities, such as gratitude and hope, we can effectively guide students to develop a positive mindset and values, improving their cognitive and problem-solving abilities and thereby enhancing their learning motivation and engagement.

### 4.5. Limitations and Implications

This study has several limitations. Firstly, the measurement of parental involvement relies solely on students’ perceived levels, which may not provide an entirely objective representation and lacks corresponding data and surveys gathered from the parents themselves. Future research could enhance the accuracy by incorporating survey data from both parents and children. Secondly, due to its cross-sectional design, this study failed to establish a strict causal relationship and draw directional conclusions; for this, future investigations could employ longitudinal designs or intervention studies to explore their directionality. A previous study found a bidirectional relationship between fathers’ educational involvement and elementary school students’ academic engagement through cross-lagged modeling of longitudinal data [66]. This also reminds us of the difference between the involvement of fathers and the involvement of mothers. In recent decades, it seems to have been conventionalized that mothers are mainly responsible for their children’s education; therefore, is it that fathers tend to be less involved in their children’s development as “economic providers” or is it the important influence of fathers in the saying “the son doesn’t teach, the father’s fault”? This is worth exploring further. Third, the sample used in this study may be lacking in representativeness, which may have influenced the accuracy and reliability of the study. Specifically, the respondents in this study came from only two schools, and given time and course scheduling constraints, this survey lacked data pertaining to grade 3 students in junior and senior high school; future research can fill this gap and appropriately improve the sample size as conditions allow and broaden the scope of the survey, e.g., gathering data from schools in different provinces. Last, different aspects of parental educational involvement may have different effects on student learning engagement, and there may be differences in the underlying mechanisms. Therefore, it is advised that future research investigations probe more deeply into the effects of intellectual involvement, emotional involvement, and behavioral involvement in parental educational involvement on students to better uncover more specific and nuanced associations.

This study profoundly reveals the centrality of parental educational involvement in the learning processes of middle school students. This finding emphasizes the indispensability of family education as an important microsystem for student learning and development, providing a solid theoretical foundation for family educational practices. By introducing gratitude and hope as mediating factors, it demonstrates the multi-level and multi-dimensional pathways through which parental educational involvement influences learning engagement, providing new perspectives and theoretical support for understanding the complex mechanisms between the two. Furthermore, educators are provided with a reference for evaluating and optimizing the effectiveness of educational interventions, enabling them to develop more targeted educational plans and counseling strategies. Parents should establish a sense of educational engagement and provide appropriate support and assistance. By optimizing the home education environment, the positive effects of parental involvement in education can be promoted, which, in turn, will enhance the learning engagement levels of students. Simultaneously, educators should focus on cultivating a positive sense of students’ gratitude and hope, and integrate gratitude and hope into family upbringing, school education, and social conditioning. Thus, the gratitude and hope of middle school students can be cultivated and enhanced to help in their academic development. By implementing these measures, we can promote the symbiotic development of family education and school education, wherein they can complement and reinforce each other and foster a productive environment for the holistic growth of students.

## 5. Conclusions

This study focuses on the association between family factors (parental education involvement), individual factors (gratitude and hope), and middle school students’ academic engagement, exploring the internal mechanisms involved. The findings demonstrate a significant positive correlation between parental educational involvement and learning engagement among middle school students. Active parental involvement in their children’s education, coupled with the provision of essential support and guidance, can effectively stimulate children’s interest and motivation in learning, thereby promoting their overall engagement. Additionally, our findings highlight the significant mediating role of gratitude and hope within this relationship. Parental educational involvement can not only make children feel cared for and supported, generating gratitude, but also help them establish a positive self-concept and goal orientation, cultivate optimism and self-confidence, and enhance hope. Increased gratitude can motivate children to value learning opportunities more and engage in learning with a more positive attitude. Elevated levels of hope will make them focus more on learning tasks and work harder to pursue their learning goals. Moreover, parental educational involvement will, by stimulating students’ gratitude, make them cherish existing learning resources and opportunities and enhance their hope for the future; thus, they will maintain lasting motivation and enthusiasm in the learning process and pursue academic achievements and future development with greater determination.

## Figures and Tables

**Table 1:** Means, standard deviations, and range of the variables in the study.

Variables	*M*	*SD*	Range	N
Gender	1.47	0.49	1–2	754
Age	14.83	1.65	11–18	754
Only child status	1.56	0.48	1–2	754
Place of birth	1.44	0.49	1–2	754
Being a class representative	1.64	0.48	1–2	754
Parental educational involvement	54.48	13.97	18–90	754
Gratitude	29.15	6.84	6–42	754
Hope	21.77	6.96	6–36	754
Learning engagement	73.15	21.39	17–119	754

Note. For gender, “1” = “male” and “2” = “female”; for only child status, “1” = “only-child”, “2” = “non-only-child”; for being a class representative, “1” = “class representative”, “2” = “non-representative”; for place of birth, “1” = “urban” and “2” = “rural”.

**Table 2:** Correlations among the main study variables.

	1	2	3	4
1. Parental educational involvement	-			
2. Gratitude	0.42 ***	-		
3. Hope	0.42 ***	0.49 ***	-	
4. Learning engagement	0.47 ***	0.51 ***	0.56 ***	-

Note. N = 754, *** *p* < 0.001, two-tailed.

**Table 3:** Regression analysis of variable relationships in chained mediation model.

Regression Equation		Overall Fitting Index	
Dependent variable	Independent variable (s)	*β*	*t*
Learning engagement			
	Parental educational involvement	0.48	14.41 ***
*R* = 0.50, *R*^2^ = 0.25, *F* = 41.34, *p* < 0.001			
Gratitude			
	Parental educational involvement	0.45	13.35 ***
*R* = 0.47, *R*^2^ = 0.22, *F* = 34.48, *p* < 0.001			
Hope			
	Parental educational involvement	0.26	7.30 ***
	Gratitude	0.37	10.86 ***
*R* = 0.56, *R*^2^ = 0.31, *F* = 48.48, *p* < 0.001			
Learning engagement			
	Parental educational involvement	0.23	6.81 ***
	Gratitude	0.23	6.83 ***
	Hope	0.35	10.41 ***
*R* = 0.66, *R*^2^ = 0.43, *F* = 70.92, *p* < 0.001			

Note. N = 754. *** *p* < 0.001.

**Table 4:** Gratitude and hope in the mediation effect analysis.

	Effect	Boot SE	Boot LLCI	Boot ULCI	Percentage of Total Effect (%)
Model pathways					
Total effect	0.48	0.03	0.41	0.54	
Direct effect	0.23	0.03	0.16	0.29	47.92%
Indirect effect	0.25	0.03	0.20	0.30	52.08%
Indirect effect 1	0.10	0.02	0.07	0.14	20.83%
Indirect effect 2	0.09	0.02	0.06	0.12	18.75%
Indirect effect 3	0.06	0.01	0.04	0.08	12.50%
Contrasts					
Ind1 minus Ind 2	0.01	0.03	−0.04	0.07	
Ind1 minus Ind 3	0.05	0.02	>0.00	0.09	
Ind2 minus Ind 3	0.03	0.02	<0.00	0.06	

Note. N = 754. LL = low limit; CI = confidence interval; UL = upper limit. Indirect effect 1: Parental educational involvement → gratitude → learning engagement; indirect effect 2: Parental educational involvement → hope → learning engagement; indirect effect 3: Parental educational involvement → gratitude → hope → learning engagement.

## Data Availability

The datasets generated during and/or analyzed during the current study are available from the corresponding author upon reasonable request.
